# Coffee Consumption and Lung Cancer Risk: The Japan Public Health Center-Based Prospective Study

**DOI:** 10.2188/jea.JE20160191

**Published:** 2018-04-05

**Authors:** Saki Narita, Eiko Saito, Norie Sawada, Taichi Shimazu, Taiki Yamaji, Motoki Iwasaki, Shizuka Sasazuki, Mitsuhiko Noda, Manami Inoue, Shoichiro Tsugane

**Affiliations:** 1 Department of Global Health Policy, Graduate School of Medicine, The University of Tokyo, Tokyo, Japan; 2 AXA Department of Health and Human Security, Graduate School of Medicine, The University of Tokyo, Tokyo, Japan; 3 Epidemiology and Prevention Group, Center for Public Health Sciences, National Cancer Center, Tokyo, Japan; 4 Department of Diabetes Research, Diabetes Research Center, National Center for Global Health and Medicine, Tokyo, Japan; 5 Department of Endocrinology and Diabetes, Saitama Medical University, Saitama, Japan

**Keywords:** coffee, lung cancer, Japan Public Health Center-based Prospective (JPHC) Study

## Abstract

**Background:**

Many epidemiological studies have indicated a positive association between coffee intake and lung cancer risk, but such findings were suggested to be confounded by smoking. Furthermore, only a few of these studies have been conducted in Asia. Here, we investigated the association between coffee intake and lung cancer risk in one of the largest prospective cohort studies in Japan.

**Methods:**

We investigated the association of coffee drinking and subsequent incidence of lung cancer among 41,727 men and 45,352 women in the Japan Public Health Center-based Prospective Study using Cox proportional hazards regression, with adjustment for potential confounders and by strata of smoking status. Coffee and other dietary intakes were assessed once at baseline with a food frequency questionnaire (FFQ).

**Results:**

During 1,481,887 person-years of follow-up between 1990 and 2011, a total of 1,668 lung cancer cases were identified. In a multivariate regression model, coffee consumption was not associated with risk of lung cancer (HR 1.16; 95% CI, 0.82–1.63; *P*_trend_ = 0.285 for men and HR 1.49; 95% CI, 0.79–2.83; *P*_trend_ = 0.942 for women). However, there was a significant increase in the risk for small cell carcinoma (HR 3.52; 95% CI, 1.49–8.28; *P*_trend_ < 0.001).

**Conclusion:**

Our prospective study suggests that habitual consumption of coffee is not associated with an increased risk of lung cancer incidence, despite observing a significant increase in the risk for small cell carcinoma.

## INTRODUCTION

Coffee is one of the most widely consumed beverages around the world, including Japan.^1^^,^^2^ Since coffee drinking is often associated with smoking, it is sometimes considered part of an unhealthy lifestyle. However, coffee is a rich source of biochemically active compounds with anti-oxidative,^3^ anti-inflammatory,^4^^,^^5^ and insulin-sensitizing effects.^6^^,^^7^ A number of studies have reported inverse associations between coffee intake and some cancers, as well as reduced mortality.^4^^,^^8^^–^^15^ Lung cancer, the world’s leading cause of cancer death,^16^ however, appears to be an exception. More than 60% of the world’s current smokers live in Asia, and the burden of lung cancer is expected to further increase in low- and middle-income countries.^17^

Epidemiological investigation into the association of coffee drinking and lung carcinogenesis has been extensive. The majority of studies have indicated positive associations. Nevertheless, adjustment for tobacco smoking remains a major issue in these studies, as it may confound the associations.^18^^–^^22^ While recent findings from a meta-analysis of 17 studies^18^ and a large prospective cohort study^19^ reported an elevated risk of lung cancer, no significant association was observed in their stratified analyses among non-smokers. In addition, very few studies have explored the association by histological subtype, despite the fact that smoking is closely linked to squamous cell and small cell carcinomas,^23^ whereas the majority of diagnoses in never smokers are adenocarcinoma.^24^ Moreover, only a single hospital-based case-control study^25^ and a few small-scale prospective cohort studies,^15^^,^^26^ with either limited information on exposure or case numbers, have been conducted in Japan.

Here, we conducted a population-based prospective cohort study to examine the association between coffee consumption and risk of lung cancer. We also investigated the association by histological subtype and comprehensive stratification of cigarette smoking.

## MATERIALS AND METHODS

### Study population

The Japan Public Health Center-based Prospective Study (JPHC Study) is an ongoing cohort study designed to explore the associations between lifestyle factors and the incidence of diseases.^27^ A total of 140,420 residents (68,722 men and 71,698 women) in 11 public health center (PHC) areas nationwide (Iwate-Ninohe, Akita-Yokote, Nagano-Saku, Tokyo-Katsushika, Okinawa-Chubu, Niigata-Nagaoka, Ibaraki-Mito, Osaka-Suita, Kochi-Chuohigashi, Nagasaki-Kamigoto, and Okinawa-Miyako) aged 40–69 years at baseline were registered between 1990 and 1994. Details of the JPHC Study have been provided elsewhere.^27^ Participants from the Tokyo-Katsushika (*n* = 7,097) and Osaka-Suita (*n* = 16,427) PHC areas were excluded due to a lack of access to data on cancer incidence. We further excluded 229 participants with non-Japanese nationality, incorrect birth date, duplicate registration, or pre-commencement emigration, and a total of 455 subjects who had late report of emigration before the start of the follow-up period or were lost to follow-up.

Of 94,917 eligible participants who returned a completed self-administered baseline questionnaire (response rate: 81.7%), those who reported a past history of cancer at any site were also not included (*n* = 1,915). After excluding subjects with extreme caloric intake (±2.5% of the distribution, *n* = 4,407) and missing data on covariates, including coffee intake and cigarette smoking (*n* = 1,516), 41,727 men and 45,352 women were included in the analytic cohort. This study was approved by the institutional review boards of the National Cancer Center (approval number: 13-021) and the University of Tokyo (approval number: 10508).

### Exposure measurement

Dietary intake, including coffee, was measured through a validated self-administered food frequency questionnaire (FFQ). In the FFQ, coffee intake was assessed using six categories based on the frequency and the amount of consumption as follows: almost never, 1–2, 3–4 times/week, and almost everyday, which was further divided into 1–2, 3–4, and ≥5 cups/day. Coffee consumption at baseline was used as an exposure in this study. Due to the small number of participants, occasional drinkers (1–2 and 3–4 times/week) were merged into one group (<1 cup/day). The validity of the questionnaire was assessed using dietary records for 28 days (1-week dietary records measured quarterly) or 14 days. Spearman rank correlation coefficients between the dietary records and coffee intake estimated from the FFQ were 0.59 for men and 0.51 for women.^28^

### Follow-up and case ascertainment

Participants were followed from the date of baseline survey (1990–1994) until the date of diagnosis of cancer, date of death, date of migration out of the study area, or the end of follow-up (December 31, 2011), whichever occurred first. Death certificates were used with the permission of the Ministry of Health, Labour and Welfare to confirm the cause of death with the International Statistical Classification of Diseases and Related Health Problems, Tenth Revision.^29^ Among the study participants, 375 (0.4%) were lost to follow-up during the follow-up period.

Identification of lung cancer cases was conducted through active patient notification from major local hospitals in each PHC area and record linkage with data from population-based cancer registries. Information on diagnosis was supplemented with information from death certificates, and 7% of cases were ascertained from death certificates only. Lung cancer cases were confirmed microscopically in 87% of cases and coded using the International Classification of Diseases for Oncology, Third Edition (C34.0–34.9).^30^ Histological subtypes included adenocarcinoma, squamous cell carcinoma, small cell carcinoma, and other types of lung carcinoma. For participants who had multiple incidences of lung cancer, only the first incidence was used.

### Statistical analysis

Cox proportional hazards regression was used to estimate hazard ratios (HRs) and 95% confidence intervals (CIs) for lung cancer incidence with coffee consumption. Participants were divided into five groups according to coffee intake, with non-drinkers as the referent group. *P*-values for trends were calculated by assigning a continuous variable from the median value for coffee intake in the regression models. In primary analyses, risk estimates for overall lung cancer incidence and histological subtypes (adenocarcinoma, squamous cell carcinoma, small cell carcinoma, and other types of lung carcinoma) were determined in separate models. To further examine the effect of residual confounding by smoking, secondary analyses were stratified by gender and detailed smoking categories, namely smoking status (never, former, and current) and smoking intensity by cumulative smoking exposure in current and former smokers by pack-years (≤19.9 or ≥20.0). Pack-years were calculated by multiplying the average number of packs smoked per day with the number of years smoked. In order to maintain sample size in scarcely populated categories, HRs were estimated by merging coffee intake into three groups as follows: almost never, <3 cups/day, or ≥3 cups/day. All covariates were assessed at baseline, and dietary intake was adjusted for total energy intake using the residual method (see Table 2 and Table 3 for details). Isoflavone was included as a confounding factor, since it was shown to have a protective effect against lung cancer risk in a previous JPHC Study.^31^ *P*-values for interaction were calculated using likelihood-ratio tests comparing Cox proportional-hazards models with and without cross-product terms for the combination of lung cancer subtypes and coffee intake or smoking status and coffee intake, with coffee as a continuous term. The proportional hazards assumption was tested with Schoenfeld residuals and shown to be insignificant. To avoid potential bias due to undiagnosed lung cancer, sensitivity analyses were conducted after excluding lung cancer cases that were diagnosed within 5 years after baseline, and these analyses produced a similar direction of associations as the main analyses. All statistical analyses were performed with Stata MP version 13.0 (StataCorp, College Station, TX, USA).

## RESULTS

During an average follow-up of 17.0 years (1,481,887 person-years), a total of 1,668 participants (1,227 men and 441 women) were newly diagnosed with lung cancer. Of histologically confirmed cases, 44% were adenocarcinoma (*n* = 729). More than 67% of the study participants reported drinking at least 1 cup/day of coffee (Table 1). Compared with non-drinkers, coffee drinkers were much more likely to smoke cigarettes. Male coffee drinkers tended to consume less alcohol, while female coffee drinkers were likely to report higher alcohol consumption. Coffee drinkers also tended to consume lower levels of fruits, vegetables, and fish, but not meat. Both male and female coffee drinkers were less likely to have diabetes.

**Table 1.** Baseline characteristics of the study participants by daily coffee consumption

Characteristics	Men (*n* = 41,727)					*P*

	No Coffee	<1 Cup/day	1–2 Cups/day	3–4 Cups/day	≥5 Cups/day	
Number of participants	13,191	12,767	10,414	3,921	1,434	
Number of lung cancer cases	403	370	296	109	49	
Age, years, mean (SD)	53.7 (7.7)	52.0 (7.7)	50.6 (7.8)	48.4 (7.3)	48.9 (7.4)	<0.001
Body mass index, kg/m^2^, mean (SD)	23.5 (2.9)	23.6 (2.8)	23.5 (2.8)	23.4 (2.9)	23.3 (3.1)	<0.001
Diabetes, %	8.6	6.3	4.7	4.1	3.9	<0.001
Current smoker, %	43.4	50.0	56.2	69.6	76.9	<0.001
Physical activity almost daily, %	6.1	4.9	4.8	4.3	5.5	<0.001
Dietary intake						
Total energy intake, kcal/day, mean (SD)	1,952 (552)	1,953 (551)	1,901 (516)	1,881 (512)	1,919 (527)	<0.001
Fruits, g/day, mean (SD)	27 (30)	27 (26)	27 (28)	26 (27)	28 (37)	<0.001
Vegetables, g/day, mean (SD)	47 (45)	45 (46)	44 (42)	40 (39)	44 (53)	<0.001
Isoflavone, mg/day, mean (SD)	18 (10)	17 (9)	16 (9)	15 (9)	15 (9)	<0.001
Alcohol consumption, g/week, mean (SD)	205 (218)	186 (209)	174 (201)	157 (197)	154 (220)	<0.001
Green tea >1 time/day, %	75.6	75.2	74.5	70.1	65.1	<0.001
Chinese tea >1 time/day, %	10.4	8.7	11.8	9.3	10.9	<0.001
Black tea >1 time/day, %	1.7	1.6	3.2	2.6	3.8	<0.001

Characteristics	Women (*n* = 45,352)					*P*

	No Coffee	<1 Cup/day	1–2 Cups/day	3–4 Cups/day	≥5 Cups/day	
Number of participants	15,202	13,554	12,573	3,155	868	
Number of lung cancer cases	163	139	103	23	13	
Age, years, mean (SD)	55.1 (7.7)	52.4 (7.8)	50.0 (7.5)	47.3 (6.6)	48.2 (7.3)	<0.001
Body mass index, kg/m^2^, mean (SD)	23.7 (3.3)	23.6 (3.1)	23.4 (3.1)	23.2 (3.0)	23.4 (3.2)	<0.001
Diabetes, %	4.9	2.5	2.0	1.3	1.3	<0.001
Current smoker, %	4.2	4.0	6.3	13.1	23.6	<0.001
Physical activity almost daily, %	5.8	4.4	4.4	3.2	5.6	<0.001
Dietary intake						
Total energy intake, kcal/day, mean (SD)	1,221 (305)	1,248 (304)	1,247 (296)	1,230 (297)	1,295 (322)	<0.001
Fruits, g/day, mean (SD)	102 (75)	100 (66)	95 (67)	89 (75)	90 (90)	<0.001
Vegetables, g/day, mean (SD)	112 (92)	106 (82)	103 (84)	92 (81)	93 (92)	<0.001
Isoflavone, mg/day, mean (SD)	26 (13)	24 (12)	23 (11)	22 (12)	20 (12)	<0.001
Alcohol consumption, g/week, mean (SD)	10 (54)	11 (54)	11 (50)	17 (82)	19 (58)	<0.001
Green tea >1 time/day, %	77.1	77.8	74.7	66.4	66.6	<0.001
Chinese tea >1 time/day, %	12.1	11.0	14.0	13.7	20.3	<0.001
Black tea >1 time/day, %	1.8	1.8	4.5	3.4	5.0	<0.001

SD denotes standard deviation. The body mass index is the weight divided by the square of the height in meters.

In models adjusted for age and PHC area, coffee consumption was associated with an increased risk of lung cancer incidence (HR 1.88; 95% CI, 1.39–2.54; *P*_trend_ < 0.001 for men and HR 2.06; 95% CI, 1.16–3.67; *P*_trend_ = 0.253 for women; comparing ≥5 cups/day with non-drinkers) (Table 2). However, participants who routinely consumed coffee also tended to be smokers, and after adjusting for smoking, the association was notably attenuated (HR 1.14; 95% CI, 0.83–1.55; *P*_trend_ = 0.405 for men and HR 1.55; 95% CI, 0.86–2.79; *P*_trend_ = 0.839 for women; comparing ≥5 cups/day with non-drinkers). Similar associations were also observed for histological subtypes. The highest hazard was seen for small cell carcinoma, with HR for the highest level of coffee intake being reduced by three-quarters after adjustment for tobacco smoking and other confounders, without significant effect modification by histological subtypes (*P*_interaction_ = 0.331 for men; *P*_interaction_ = 0.169 for women). In models separately analyzed by sex, hazard ratios comparing ≥5 cups/day with non-drinkers for small cell carcinoma were higher for female coffee drinkers than male drinkers (HR 4.07; 95% CI, 0.74–22.49; *P*_trend_ = 0.033 for women and HR 2.82; 95% CI, 1.00–7.94; *P*_trend_ = 0.005 for men) (eTable 1). No other clear gender differences were observed in the histological analyses.

**Table 2.** Hazard ratios (95% confidence intervals) for lung cancer incidence by daily coffee consumption

	Coffee intake					*P*-trend	*P*-interaction

	No Coffee	<1 Cup/day	1–2 Cups/day	3–4 Cups/day	≥5 Cups/day		
**Lung cancer (all)**							
*Men*							0.331
Number of cases	403	370	296	109	49	—	—
Age, area	1.00	1.07 (0.93–1.23)	1.27 (1.09–1.48)	1.54 (1.24–1.91)	1.88 (1.39–2.54)	<0.001	—
Age, area, smoking	1.00	0.95 (0.82–1.10)	1.05 (0.89–1.23)	1.02 (0.82–1.28)	1.14 (0.83–1.55)	0.405	—
Multivariate-adjusted	1.00	1.03 (0.88–1.22)	1.11 (0.93–1.22)	1.05 (0.82–1.34)	1.16 (0.82–1.63)	0.285	—
*Women*							0.169
Number of cases	163	139	103	23	13	—	—
Age, area	1.00	1.08 (0.86–1.36)	1.00 (0.77–1.30)	1.08 (0.69–1.70)	2.06 (1.16–3.67)	0.253	—
Age, area, smoking	1.00	1.08 (0.86–1.36)	0.95 (0.73–1.24)	0.90 (0.56–1.43)	1.55 (0.86–2.79)	0.839	—
Multivariate-adjusted	1.00	1.06 (0.82–1.36)	0.94 (0.70–1.25)	0.90 (0.55–1.48)	1.49 (0.79–2.83)	0.942	—
**Adenocarcinoma**							
Number of cases	266	216	176	46	25	—	—
Age, gender, area	1.00	0.97 (0.81–1.16)	1.10 (0.90–1.33)	1.06 (0.77–1.46)	1.66 (1.09–2.51)	0.087	—
Age, gender, area, smoking	1.00	0.91 (0.76–1.09)	0.97 (0.80–1.19)	0.83 (0.59–1.16)	1.21 (0.79–1.86)	0.829	—
Multivariate-adjusted	1.00	0.95 (0.78–1.16)	1.01 (0.81–1.26)	0.80 (0.56–1.16)	1.13 (0.70–1.83)	0.778	—
**Squamous cell carcinoma**							
Number of cases	107	106	76	30	16	—	—
Age, gender, area	1.00	1.20 (0.91–1.57)	1.31 (0.97–1.77)	1.80 (1.19–2.73)	2.55 (1.50–4.35)	<0.001	—
Age, gender, area, smoking	1.00	1.06 (0.80–1.40)	1.06 (0.78–1.45)	1.10 (0.71–1.70)	1.45 (0.84–2.49)	0.303	—
Multivariate-adjusted	1.00	1.14 (0.83–1.55)	1.10 (0.78–1.55)	1.04 (0.63–1.69)	1.24 (0.66–2.32)	0.578	—
**Small cell carcinoma**							
Number of cases	28	61	37	21	9	—	—
Age, gender, area	1.00	2.60 (1.66–4.08)	2.28 (1.38–3.77)	4.50 (2.51–8.06)	5.39 (2.51–11.58)	<0.001	—
Age, gender, area, smoking	1.00	2.47 (1.56–3.93)	1.88 (1.12–3.16)	2.83 (1.54–5.17)	2.62 (1.16–5.90)	0.003	—
Multivariate-adjusted	1.00	2.51 (1.47–4.30)	2.35 (1.33–4.17)	3.48 (1.79–6.73)	3.52 (1.49–8.28)	<0.001	—
**Other**							
Number of cases	165	126	110	35	12	—	—
Age, gender, area	1.00	0.93 (0.74–1.17)	1.17 (0.92–1.51)	1.33 (0.92–1.94)	1.27 (0.70–2.29)	0.072	—
Age, gender, area, smoking	1.00	0.87 (0.68–1.10)	1.00 (0.77–1.29)	0.94 (0.64–1.38)	0.83 (0.46–1.38)	0.707	—
Multivariate-adjusted	1.00	0.94 (0.72–1.23)	1.00 (0.75–1.35)	0.99 (0.65–1.50)	1.02 (0.55–1.88)	0.958	—

Multivariate analyses were adjusted for the following factors at baseline: age (continuous); gender; PHC area; body-mass index (<18.5, 18.5–24.9, 25.0–29.9, and ≥30 kg/m^2^); smoking status (never, former: <10, 10–19, and ≥20 years of smoking cessation, current: 1–19, 20–29, 30–39, 40–49, 50–59, ≥60 pack-years); physical activity (almost never, <3 days/month, 1–2 days/week, 3–4 days/week, and almost everyday); alcohol consumption (never/former, <1 time/week, <23, 23–45, 46–68, 69–91, and >92 g of ethanol/day); consumption of green tea, Chinese tea, and black tea (almost never, <1 time/week, and >1 cup/day); energy-adjusted intake of fruit, vegetables, and isoflavone (continuous).

Approximately half of male participants were current smokers, whereas 93% of female participants were never-smokers (Table 3). In stratified analyses by smoking strata, current and former male smokers were examined by pack-years. A higher level of coffee intake among male heavy smokers who had ≥20.0 pack-years of tobacco exposure was associated with an increased risk of lung cancer (HR 1.30; 95% CI, 1.04–1.63; *P*_trend_ = 0.014, comparing ≥3 cups/day with non-drinkers), whereas no substantial effect modification by smoking status was observed in both men and women (*P*_interaction_ = 0.266 for men and *P*_interaction_ = 0.243 for women). While tea is also known to have free radical scavenging activity and chemopreventive effects on lung cancer,^32^^,^^33^ a separate analysis on tea, including green tea, Chinese (oolong) tea, and black tea, and lung cancer did not find a significant association (eTable 2).

**Table 3.** Subgroup analysis by smoking status: hazard ratios (95% confidence intervals) for lung cancer incidence by daily coffee consumption

	All Participants	Coffee intake			*P*-trend	*P*-interaction

		No Coffee	<3 Cups/day	≥3 Cups/day		
**Men**						0.266
*Never-smokers*						
Number of participants	10,138	3,804	5,615	719	—	—
Number of cases	87	40	40	7	—	—
Age, area		1.00	0.75 (0.48–1.17)	1.20 (0.53–2.71)	0.587	—
Multivariate-adjusted		1.00	0.81 (0.49–1.33)	1.41 (0.58–3.46)	0.940	—
*Former smokers*						
Number of participants	9,789	3,660	5,323	806	—	—
Number of cases	188	75	105	8	—	—
Age, area		1.00	1.11 (0.82–1.50)	0.85 (0.41–1.78)	0.802	—
Multivariate-adjusted		1.00	1.25 (0.90–1.74)	0.88 (0.40–1.95)	0.461	—
*Current smokers*						
Number of participants	21,800	5,727	12,243	3,830	—	—
Number of cases	952	288	521	143	—	—
Age, area		1.00	1.04 (0.90–1.20)	1.18 (0.96–1.46)	0.155	—
Multivariate-adjusted		1.00	1.12 (0.95–1.32)	1.24 (0.99–1.57)	0.058	—
*Light smokers: pack-years ≤19.9*^a^						
Number of participants	7,846	2,654	4,446	746	—	—
Number of cases	100	38	58	4	—	—
Age, area		1.00	1.14 (0.75–1.73)	0.61 (0.21–1.73)	0.876	—
Multivariate-adjusted		1.00	1.35 (0.83–2.18)	0.92 (0.31–2.68)	0.500	—
*Heavy smokers: pack-years ≥20.0*^a^						
Number of participants	23,743	6,733	13,120	3,890	—	—
Number of cases	1,040	325	568	147	—	—
Age, area		1.00	1.08 (0.94–1.24)	1.29 (1.05–1.58)	0.023	—
Multivariate-adjusted		1.00	1.16 (0.99–1.35)	1.30 (1.04–1.63)	0.014	—

**Women**						0.243
*Never-smokers*						
Number of participants	42,155	14,374	24,458	3,323	—	—
Number of cases	375	147	204	24	—	—
Age, area		1.00	1.01 (0.81–1.25)	1.14 (0.72–1.79)	0.716	—
Multivariate-adjusted		1.00	1.00 (0.79–1.28)	1.13 (0.70–1.84)	0.759	—
*Current smokers*						
Number of participants	2,600	645	1,337	618	—	—
Number of cases	61	14	35	12	—	—
Age, area		1.00	1.42 (0.76–2.67)	1.39 (0.61–3.14)	0.380	—
Multivariate-adjusted		1.00	1.27 (0.66–2.45)	1.38 (0.58–3.28)	0.441	—

Multivariate analyses were adjusted for the following factors at baseline: age (continuous); gender; PHC area; body-mass index (<18.5, 18.5–24.9, 25.0–29.9, and ≥30 kg/m^2^); smoking status (never, former: <10, 10–19, and ≥20 years of smoking cessation, current: 1–19, 20–29, 30–39, 40–49, 50–59, ≥60 pack-years); physical activity (almost never, <3 days/month, 1–2 days/week, 3–4 days/week, and almost everyday); alcohol consumption (never/former, <1 time/week, <23, 23–45, 46–68, 69–91, and >92 g of ethanol/day); consumption of green tea, Chinese tea, and black tea (almost never, <1 time/week, and >1 cup/day); energy-adjusted intake of fruit, vegetables, and isoflavone (continuous).

^a^Model included current and former smokers. Pack-years is the number of cigarette packs (assuming 20 cigarettes per pack) smoked per day, multiplied by years of consumption.

## DISCUSSION

In this prospective Japanese cohort study, our overall results suggested that coffee consumption is not significantly associated with an increased risk of lung cancer, after adjustment for smoking and other potential confounders. Residual confounding by smoking is a major concern in lung cancer research; the majority of our present coffee drinkers tended to be smokers, and the adjustment for cigarette smoking might have been incomplete, which would have attenuated our results. The significant positive association among heavy smokers might be explained via the residual confounding by smoking. An updated meta-analysis of five cohort and 12 case-control studies indicated that recently published studies consistently reported a positive association of coffee intake with lung cancer incidence, despite a reduction in the risk of other types of cancers, including prostate, bladder, colorectal, and liver cancers.^18^ While the positive associations were consistent not only in case-control but also prospective cohort studies, many were detected among smokers, so cautious interpretation was recommended.^19^^,^^34^^,^^35^ Furthermore, the possibility of passive inhalation of cigarette smoke by non-smokers should also be considered. Presence or absence of passive smoking at home and work was included in a separate model in an attempt to assess environmental exposure, although the period and intensity of exposure could not be measured due to data unavailability. While this analysis may have only partially captured the influence of passive smoking, the risk estimates were not substantially altered (data not shown). Null associations between coffee intake and lung cancer risk observed among non-smokers in the present study were consistent with the majority of recent studies.^18^^,^^19^

In the stratified analyses by subsite of lung cancer, an increased risk of small cell carcinoma was found among coffee drinkers. This finding was congruous with a recent large-scale prospective cohort study in the United States wherein coffee intake was positively associated with small cell carcinoma.^19^ Smoking has been closely linked with small cell and squamous cell carcinomas in previous studies,^23^^,^^36^ and we found a similar association in the present study, with approximately 92% (126/137 cases) of small cell carcinoma cases in men observed among heavy smokers with ≥20.0 pack-years (data not shown). The steep increase in the risk of these subtypes of lung carcinoma could be explained via residual confounding by smoking. The oncogenic mechanism of this association has yet to be elucidated, but one possible pathway might be through the activity of TP53 and RB1, the tumor suppressor genes that play a crucial role in the oncogenesis of small cell carcinoma.^37^ Contrary to its chemopreventive components,^3^^–^^5^^,^^38^^,^^39^ coffee has been recently discovered to contain gallic acid and pyrogallol, which are considered responsible for DNA-damaging activities involving TP53.^40^ Further analysis is required to examine the potential impact of coffee intake on the etiology of subtypes of lung carcinogenesis and to investigate the possibility of chance findings, as there were only modest numbers of cases in some of our subanalyses.

Some limitations of the present study include the unavailability of information on types of coffee (caffeinated or decaffeinated), preparation methods (eg, drip, instant, or canned), duration of coffee consumption, and cumulative intake of coffee and caffeine, although consumption of decaffeinated coffee is infrequent in Japan. A recent prospective cohort study that investigated the effect of caffeine intake on cancer risk reported a null association with lung cancer.^21^ While coffee drinking is considered to be relatively stable over time, coffee intake was evaluated at baseline using a single self-reported measurement, and the possibility of misclassification cannot be ruled out. However, such misclassification due to fluctuation in habitual coffee consumption during the follow-up period would likely have occurred randomly and nondifferentially and might have attenuated the results towards null. We also excluded participants who reported a history of cancer at any site and conducted a sensitivity analysis excluding participants with newly diagnosed lung cancer, which would refute a possibility of a bias from ongoing or undiagnosed illnesses. Further, cumulative exposure to cigarette smoking was taken into consideration as pack-years, but more detailed data on smoking, such as depth of inhalation or nicotine dose, was unavailable.

Despite these limitations, this is the first population-based prospective cohort study to investigate the association between coffee intake and lung cancer incidence in Japan. The main strengths of our study include its high response rate (81.7%), long follow-up period and negligible number of lost participants (0.4%), and minimal recall bias due to its prospective design. Misclassification of lung cancer cases was also unlikely because of the rate of microscopic diagnosis and limited reliance on death certificate notification.

In summary, we found that a higher level of coffee intake was not associated with an increased risk of lung cancer, although we did observe an elevated risk of small cell carcinoma. This positive association was substantially attenuated after adjusting for cigarette smoking. Further evidence is needed to determine the associations with subtypes of lung cancer. Future studies that capture more detailed quantitative information on tobacco exposure would help clarify the impact of residual confounding on lung carcinogenesis.

## APPENDIX A. SUPPLEMENTARY DATA

eTable 1. Hazard ratios (95% confidence intervals) for lung cancer incidence by daily coffee consumption: sub-analysis by gender

eTable 2. Hazard ratios (95% confidence intervals) for lung cancer incidence by daily tea (green, oolong, and black tea) consumption

## References

[1] Iso H, Date C, Wakai K, Fukui M, Tamakoshi A; JACC Study Group The relationship between green tea and total caffeine intake and risk for self-reported type 2 diabetes among Japanese adults. Ann Intern Med. 2006;144(8):554–562. 10.7326/0003-4819-144-8-200604180-00005 16618952

[2] Food and Agriculture Organization. *Medium-term prospects for agricultural commodities: projections to the year 2030*. Rome, Italy: Food and Agriculture Organization of the United Nations; 2003.

[3] Ludwig IA, Clifford MN, Lean ME, Ashihara H, Crozier A Coffee: biochemistry and potential impact on health. Food Funct. 2014;5(8):1695–1717. 10.1039/C4FO00042K 24671262

[4] Lopez-Garcia E Long-term coffee consumption associated with reduced risk of total and cause-specific mortality. Evid Based Med. 2013;18(3):116–117. 10.1136/eb-2012-100878 22923704

[5] Shen T, Park YC, Kim SH, Lee J, Cho JY Nuclear factor-kappaB/signal transducers and activators of transcription-1-mediated inflammatory responses in lipopolysaccharide-activated macrophages are a major inhibitory target of kahweol, a coffee diterpene. Biol Pharm Bull. 2010;33(7):1159–1164. 10.1248/bpb.33.1159 20606307

[6] Huxley R, Lee CM, Barzi F, Coffee, decaffeinated coffee, and tea consumption in relation to incident type 2 diabetes mellitus: a systematic review with meta-analysis. Arch Intern Med. 2009;169(22):2053–2063. 10.1001/archinternmed.2009.439 20008687

[7] van Dam RM, Feskens EJ Coffee consumption and risk of type 2 diabetes mellitus. Lancet. 2002;360(9344):1477–1478. 10.1016/S0140-6736(02)11436-X 12433517

[8] Saito E, Inoue M, Sawada N, Association of coffee intake with total and cause-specific mortality in a Japanese population: the Japan Public Health Center-based Prospective Study. Am J Clin Nutr. 2015;101(5):1029–1037. 10.3945/ajcn.114.104273 25762807

[9] Ding M, Satija A, Bhupathiraju SN, Association of coffee consumption with total and cause-specific mortality in 3 large prospective cohorts. Circulation. 2015;132(24):2305–2315. 10.1161/CIRCULATIONAHA.115.017341 26572796 PMC4679527

[10] Loftfield E, Freedman ND, Graubard BI, Association of coffee consumption with overall and cause-specific mortality in a large US prospective cohort study. Am J Epidemiol. 2015;182(12):1010–1022. 10.1093/aje/kwv146 26614599 PMC5875735

[11] Crippa A, Discacciati A, Larsson SC, Wolk A, Orsini N Coffee consumption and mortality from all causes, cardiovascular disease, and cancer: a dose-response meta-analysis. Am J Epidemiol. 2014;180(8):763–775. 10.1093/aje/kwu194 25156996

[12] Freedman ND, Park Y, Abnet CC, Hollenbeck AR, Sinha R Association of coffee drinking with total and cause-specific mortality. N Engl J Med. 2012;366(20):1891–1904. 10.1056/NEJMoa1112010 22591295 PMC3439152

[13] Happonen P, Läärä E, Hiltunen L, Luukinen H Coffee consumption and mortality in a 14-year follow-up of an elderly northern Finnish population. Br J Nutr. 2008;99(6):1354–1361. 10.1017/S0007114507871650 18062826

[14] Paganini-Hill A, Kawas CH, Corrada MM Non-alcoholic beverage and caffeine consumption and mortality: the Leisure World Cohort Study. Prev Med. 2007;44(4):305–310. 10.1016/j.ypmed.2006.12.011 17275898 PMC2034362

[15] Khan MM, Goto R, Kobayashi K, Dietary habits and cancer mortality among middle aged and older Japanese living in Hokkaido, Japan by cancer site and sex. Asian Pac J Cancer Prev. 2004;5:58–65. 15075007

[16] Ferlay J, Soerjomataram I, Dikshit R, Cancer incidence and mortality worldwide: sources, methods and major patterns in GLOBOCAN 2012. Int J Cancer. 2015;136(5):E359–E386. 10.1002/ijc.29210 25220842

[17] Islami F, Stoklosa M, Drope J, Jemal A Global and regional patterns of tobacco smoking and tobacco control policies. Eur Urol Focus. 2015;1(1):3–16. 10.1016/j.euf.2014.10.001 28723352

[18] Xie Y, Qin J, Nan G, Huang S, Wang Z, Su Y Coffee consumption and the risk of lung cancer: an updated meta-analysis of epidemiological studies. Eur J Clin Nutr. 2016;70(2):199–206. 10.1038/ejcn.2015.96 26081490

[19] Guertin KA, Freedman ND, Loftfield E, Graubard BI, Caporaso NE, Sinha R Coffee consumption and incidence of lung cancer in the NIH-AARP Diet and Health Study. Int J Epidemiol. 2016;45(3):929–939. 10.1093/ije/dyv104 26082405 PMC5005936

[20] Sanikini H, Radoï L, Menvielle G, Coffee consumption and risk of lung cancer: the ICARE study. Eur J Epidemiol. 2015;30(1):81–85. 10.1007/s10654-014-9976-2 25504015

[21] Hashibe M, Galeone C, Buys SS, Coffee, tea, caffeine intake, and the risk of cancer in the PLCO cohort. Br J Cancer. 2015;113(5):809–816. 10.1038/bjc.2015.276 26291054 PMC4559834

[22] Tang N, Wu Y, Ma J, Wang B, Yu R Coffee consumption and risk of lung cancer: a meta-analysis. Lung Cancer. 2010;67(1):17–22. 10.1016/j.lungcan.2009.03.012 19362749

[23] National Cancer Center. *Lung Cancer*. Tokyo, Japan: National Cancer Center; 2014.

[24] Subramanian J, Govindan R Lung cancer in never smokers: a review. J Clin Oncol. 2007;25(5):561–570. 10.1200/JCO.2006.06.8015 17290066

[25] Takezaki T, Hirose K, Inoue M, Dietary factors and lung cancer risk in Japanese: with special reference to fish consumption and adenocarcinomas. Br J Cancer. 2001;84(9):1199–1206. 10.1054/bjoc.2001.1722 11336471 PMC2363884

[26] Fu YY, Takezaki T, Tajima K [Risk factors of lung cancer--follow-up studies in Nagoya Japan]. Zhonghua Liu Xing Bing Xue Za Zhi. 1997;18(6):328–330. 9812533

[27] Tsugane S, Sawada N The JPHC study: design and some findings on the typical Japanese diet. Jpn J Clin Oncol. 2014;44(9):777–782. 10.1093/jjco/hyu096 25104790

[28] Inoue M, Kurahashi N, Iwasaki M, ; Japan Public Health Center-Based Prospective Study Group Effect of coffee and green tea consumption on the risk of liver cancer: cohort analysis by hepatitis virus infection status. Cancer Epidemiol Biomarkers Prev. 2009;18(6):1746–1753. 10.1158/1055-9965.EPI-08-0923 19505908

[29] World Health Organization. *International Statistical Classification of Diseases and Related Health Problems, Tenth Revision*. Geneva, Switzerland: WHO; 1990. 3376487

[30] World Health Organization. *International Classification of Diseases for Oncology*. Geneva: WHO; 2000.

[31] Shimazu T, Inoue M, Sasazuki S, ; Japan Public Health Center-based Prospective Study Group Isoflavone intake and risk of lung cancer: a prospective cohort study in Japan. Am J Clin Nutr. 2010;91(3):722–728. 10.3945/ajcn.2009.28161 20071645

[32] Tang N, Wu Y, Zhou B, Wang B, Yu R Green tea, black tea consumption and risk of lung cancer: a meta-analysis. Lung Cancer. 2009;65(3):274–283. 10.1016/j.lungcan.2008.12.002 19128856

[33] Pham-Huy NLA, He H, Pham-Huy C Green tea and health: an overview. J Food Agric Environ. 2008;6(1):6–13.

[34] Bae JM, Li ZM, Shin MH, Kim DH, Lee MS, Ahn YO Pulmonary tuberculosis and lung cancer risk in current smokers: the Seoul Male Cancer Cohort Study. J Korean Med Sci. 2013;28(6):896–900. 10.3346/jkms.2013.28.6.896 23772155 PMC3678007

[35] Baker JA, McCann SE, Reid ME, Nowell S, Beehler GP, Moysich KB Associations between black tea and coffee consumption and risk of lung cancer among current and former smokers. Nutr Cancer. 2005;52(1):15–21. 10.1207/s15327914nc5201_2 16090999

[36] Wakai K, Inoue M, Mizoue T, ; Research Group for the Development and Evaluation of Cancer Prevention Strategies in Japan Tobacco smoking and lung cancer risk: an evaluation based on a systematic review of epidemiological evidence among the Japanese population. Jpn J Clin Oncol. 2006;36(5):309–324. 10.1093/jjco/hyl025 16735374

[37] George J, Lim JS, Jang SJ, Comprehensive genomic profiles of small cell lung cancer. Nature. 2015;524(7563):47–53. 10.1038/nature14664 26168399 PMC4861069

[38] Bakuradze T, Lang R, Hofmann T, Antioxidant effectiveness of coffee extracts and selected constituents in cell-free systems and human colon cell lines. Mol Nutr Food Res. 2010;54(12):1734–1743. 10.1002/mnfr.201000147 20589861

[39] Glei M, Kirmse A, Habermann N, Persin C, Pool-Zobel BL Bread enriched with green coffee extract has chemoprotective and antigenotoxic activities in human cells. Nutr Cancer. 2006;56(2):182–192. 10.1207/s15327914nc5602_9 17474864

[40] Hossain MZ, Gilbert SF, Patel K, Ghosh S, Bhunia AK, Kern SE Biological clues to potent DNA-damaging activities in food and flavoring. Food Chem Toxicol. 2013;55:557–567. 10.1016/j.fct.2013.01.058 23402862 PMC3608747

