# Supplementary material for: Coffee Consumption and Lung Cancer Risk: The Japan Public Health Center-Based Prospective Study
Source: J Epidemiol. 2018 Apr 5;28(4):207–13. doi: 10.2188/jea.JE20160191 (PMC5865012; doi:10.2188/jea.JE20160191)
Supplement: Supplementary file 1 [file je-28-207-s001.pdf]

eTable 1. Hazard ratios (95% confidence intervals) for lung cancer incidence by daily coffee consumption: sub-analysis by gender

Lung cancer	Coffee intake					*P*-trend
	No Coffee	<1 Cup/day	1–2 Cups/day	3–4 Cups/day	≥5 Cups/day	
**Adenocarcinoma**						
Men						
Number of cases	146	115	109	32	17	—
Age, area	1.00	0.89 (0.69–1.13)	1.21 (0.94–1.56)	1.12 (0.76–1.66)	1.63 (0.98–2.70)	0.048
Age, area, smoking	1.00	0.77 (0.60–0.99)	1.01 (0.78–1.31)	0.78 (0.52–1.17)	1.06 (0.62–1.80)	0.770
Multivariate-adjusted	1.00	0.84 (0.64–1.11)	1.09 (0.82–1.44)	0.76 (0.48–1.20)	1.00 (0.55–1.81)	0.831
Women						
Number of cases	120	101	67	14	8	—
Age, area	1.00	1.06 (0.81–1.38)	0.90 (0.65–1.22)	0.89 (0.50–1.58)	1.72 (0.83–3.56)	0.975
Age, area, smoking	1.00	1.06 (0.81–1.39)	0.86 (0.62–1.18)	0.87 (0.50–1.55)	1.67 (0.80–3.48)	0.818
Multivariate-adjusted	1.00	1.07 (0.79–1.43)	0.84 (0.59–1.20)	0.85 (0.46–1.60)	1.48 (0.63–3.45)	0.659
**Squamous cell carcinoma**						
Men						
Number of cases	99	95	68	29	15	—
Age, area	1.00	1.14 (0.86–1.52)	1.26 (0.92–1.73)	1.83 (1.20–2.80)	2.49 (1.44–4.32)	<0.001
Age, area, smoking	1.00	1.00 (0.75–1.35)	1.01 (0.73–1.41)	1.15 (0.74–1.79)	1.41 (0.81–2.48)	0.322
Multivariate-adjusted	1.00	1.11 (0.80–1.53)	1.08 (0.75–1.55)	1.10 (0.67–1.81)	1.19 (0.62–2.30)	0.563
Women						
Number of cases	8	11	8	1	1	—
Age, area	1.00	1.80 (0.72–4.52)	1.67 (0.59–4.68)	1.03 (0.12–8.69)	3.42 (0.41–28.46)	0.316
Age, area, smoking	1.00	1.62 (0.63–4.12)	1.47 (0.53–4.11)	NA	1.28 (0.15–11.05)	0.914
Multivariate-adjusted	1.00	1.42 (0.49–4.09)	1.29 (0.40–4.13)	NA	1.16 (0.13–10.69)	0.763
**Small cell carcinoma**						
Men						
Number of cases	24	58	32	17	6	—
Age, area	1.00	2.84 (1.76–4.57)	2.32 (1.36–3.98)	4.05 (2.14–7.66)	4.10 (1.65–10.14)	<0.001
Age, area, smoking	1.00	2.72 (1.66–4.45)	1.94 (1.11–3.39)	2.60 (1.34–5.06)	2.00 (0.74–5.38)	0.026
Multivariate-adjusted	1.00	2.91 (1.62–5.22)	2.49 (1.32–4.69)	3.25 (1.55–6.81)	2.82 (1.00–7.94)	0.005
Women						
Number of cases	4	3	5	4	3	—
Age, area	1.00	0.92 (0.20–4.13)	1.68 (0.43–6.61)	6.86 (1.55–30.33)	15.24 (3.17–73.24)	<0.001
Age, area, smoking	1.00	0.91 (0.20–4.09)	1.33 (0.34–5.17)	3.40 (0.77–15.07)	4.34 (0.86–21.96)	0.040
Multivariate-adjusted	1.00	0.55 (0.08–3.10)	1.16 (0.29–4.68)	4.30 (0.94–19.80)	4.07 (0.74–22.49)	0.033
**Other**						
Men						
Number of cases	134	102	87	31	11	—
Age, area	1.00	0.90 (0.66–1.17)	1.14 (0.87–1.51)	1.37 (0.92–2.05)	1.32 (0.71–2.46)	0.085
Age, area, smoking	1.00	0.82 (0.63–1.07)	0.95 (0.72–1.27)	0.93 (0.62–1.41)	0.85 (0.46–1.60)	0.665
Multivariate-adjusted	1.00	0.92 (0.68–1.24)	0.94 (0.68–1.31)	0.97 (0.62–1.53)	1.06 (0.56–2.02)	0.947
Women						
Number of cases	31	24	23	4	1	—
Age, area	1.00	1.00 (0.58–1.71)	1.18 (0.67–2.08)	1.00 (0.34–2.94)	0.87 (0.12–6.46)	0.766
Age, area, smoking	1.00	1.05 (0.61–1.81)	1.12 (0.63–2.00)	0.79 (0.27–2.37)	0.51 (0.07–3.92)	0.798
Multivariate-adjusted	1.00	0.97 (0.53–1.77)	1.14 (0.61–2.14)	0.83 (0.27–2.55)	0.67 (0.09–5.12)	0.937

Multivariate analyses were adjusted for the following factors at baseline: age (continuous); gender; PHC area; body-mass index (<18.5, 18.5–24.9, 25.0–29.9, and ≥30 kg/m^2); smoking status (never, former: <10, 10–19, and ≥20 years of smoking cessation, current: 1–19, 20–29, 30–39, 40–49, 50–59, ≥ 60 pack-years); physical activity (almost never, <3 days/month, 1–2 days/week, 3–4 days/week, and almost everyday); alcohol consumption (never/former, <1 time/week, <23, 23–45, 46–68, 69–91, and >92 g of ethanol/day); consumption of green tea, Chinese tea, and black tea (almost never, <1 time/week, and >1 cup/day); energy-adjusted intake of fruit, vegetables, and isoflavone (continuous).

eTable 2. Hazard ratios (95% confidence intervals) for lung cancer incidence by daily tea (green, oolong, and black tea) consumption

Lung cancer	Tea intake			*P*-trend
	No tea	<3 Cup/day	≥3 Cups/day	
**Green tea**				
Number of cases	173	160	1,318	—
Age, area	1.00	0.90 (0.72–1.11)	1.00 (0.85–1.18)	0.687
Multivariate-adjusted	1.00	1.01 (0.79–1.30)	1.09 (0.90–1.32)	0.928
**Chinese (oolong) tea**				
Number of cases	1,120	288	154	—
Age, area	1.00	0.86 (0.76–0.98)	0.98 (0.82–1.16)	0.235
Multivariate-adjusted	1.00	0.87 (0.76–0.99)	0.99 (0.83–1.18)	0.310
**Black tea**				
Number of cases	1,392	212	35	—
Age, area	1.00	0.90 (0.78–1.04)	0.98 (0.70–1.37)	0.253
Multivariate-adjusted	1.00	0.92 (0.80–1.07)	1.02 (0.72–1.44)	0.476

Multivariate analyses were adjusted for the following factors at baseline: age (continuous); gender; PHC area; body-mass index (<18.5, 18.5–24.9, 25.0–29.9, and ≥30 kg/m^2); smoking status (never, former: <10, 10–19, and ≥20 years of smoking cessation, current: 1–19, 20–29, 30–39, 40–49, 50–59, ≥60 pack-years); physical activity (almost never, <3 days/month, 1–2 days/week, 3–4 days/week, and almost everyday); alcohol consumption (never/former, <1 time/week, <23, 23–45, 46–68, 69–91, and >92 g of ethanol/day); coffee intake (almost never, <1 cup/day, 1–2 cups/day, 3–4 cups/day, ≥5 cups/day); energy-adjusted intake of fruit, vegetables, and isoflavone (continuous).
